# 4-Hydrazino-1-methyl­pyrazolo[3,4-*d*]pyrimidine

**DOI:** 10.1107/S1600536809023952

**Published:** 2009-07-01

**Authors:** Anton V. Dolzhenko, Giorgia Pastorin, Anna V. Dolzhenko, Geok Kheng Tan, Lip Lin Koh

**Affiliations:** a Department of Pharmacy, Faculty of Science, National University of Singapore, 18 Science Drive 4, Singapore 117543, Singapore; b Department of Chemistry, Faculty of Science, National University of Singapore, 3 Science Drive 3, Singapore 117543, Singapore

## Abstract

The title compound, C_6_H_8_N_6_, crystallizes as an N—H⋯N hydrogen-bond-linked dimer of two almost identical mol­ecules in the asymmetric unit. Both of the mol­ecules are almost planar (rms deviations of 0.0186 and 0.0296 Å in the two molecules) and their hydrazino groups are turned towards the pyrazole rings. The dimers are arranged into chains *via* inter­molecular N—H⋯N hydrogen bonds between the hydrazino groups and the N atoms of the pyrimidine rings of both types of the mol­ecules, linking the mol­ecules into a *C*(7) graph-set motif along [100]. The methyl groups and the N atoms of the pyrazole rings form weak C—H⋯N hydrogen bonds, which connect chains of the dimers in a *C*(4) motif parallel to [100].

## Related literature

For recent reviews on the synthesis and biological activity of pyrazolo[3,4-*d*]pyrimidines, see: Caravatti *et al.* (2001 ▶); Dang (2002 ▶); Schenone *et al.* (2007 ▶); Schenone *et al.* (2008 ▶). The synthesis of the title compound was performed according to the procedure reported by Taylor & Loeffler (1960 ▶). For the crystal structure of 1-methyl-4-(2-methyl­hydrazino)pyrazolo[3,4-*d*]pyrimidine, see: Hosmane *et al.* (1988 ▶). For the graph-set analysis of hydrogen bonding, see: Bernstein *et al.* (1995 ▶).
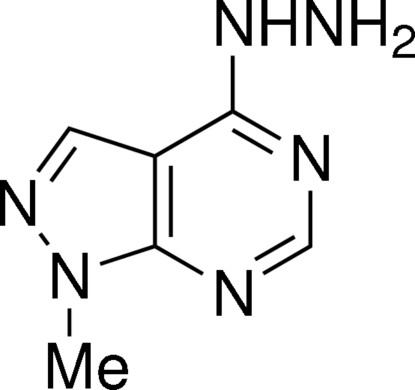


## Experimental

### 

#### Crystal data

C_6_H_8_N_6_
*M* *_r_* = 164.18
Orthorhombic, 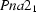

*a* = 14.086 (4) Å
*b* = 3.8756 (12) Å
*c* = 27.271 (8) Å
*V* = 1488.8 (8) Å^3^
*Z* = 8
Mo *K*α radiation
μ = 0.10 mm^−1^
*T* = 223 K
0.58 × 0.26 × 0.10 mm

#### Data collection

Bruker SMART APEX CCD diffractometer
Absorption correction: multi-scan (*SADABS*; Sheldrick, 2001 ▶) *T*_min_ = 0.943, *T*_max_ = 0.990
8939 measured reflections
1715 independent reflections
1652 reflections with *I* > 2σ(*I*)
*R*_int_ = 0.051

#### Refinement

*R*[*F* ^2^ > 2σ(*F* ^2^)] = 0.065
*wR*(*F* ^2^) = 0.159
*S* = 1.21
1715 reflections
243 parameters
1 restraint
H atoms treated by a mixture of independent and constrained refinement
Δρ_max_ = 0.29 e Å^−3^
Δρ_min_ = −0.28 e Å^−3^

### 

Data collection: *SMART* (Bruker, 2001 ▶); cell refinement: *SAINT* (Bruker, 2001 ▶); data reduction: *SAINT*; program(s) used to solve structure: *SHELXS97* (Sheldrick, 2008 ▶); program(s) used to refine structure: *SHELXL97* (Sheldrick, 2008 ▶); molecular graphics: *SHELXTL* (Sheldrick, 2008 ▶); software used to prepare material for publication: *SHELXTL*.

## Supplementary Material

Crystal structure: contains datablocks I, global. DOI: 10.1107/S1600536809023952/jh2082sup1.cif

Structure factors: contains datablocks I. DOI: 10.1107/S1600536809023952/jh2082Isup2.hkl

Additional supplementary materials:  crystallographic information; 3D view; checkCIF report

## Figures and Tables

**Table 1:** Hydrogen-bond geometry (Å, °)

*D*—H⋯*A*	*D*—H	H⋯*A*	*D*⋯*A*	*D*—H⋯*A*
C12—H12*B*⋯N1^i^	0.97	2.65	3.297 (7)	124
C6—H6*C*⋯N7^ii^	0.97	2.54	3.410 (7)	150
N11—H11*N*⋯N4	0.85 (6)	2.11 (6)	2.948 (6)	170 (5)
N5—H5*N*⋯N10	0.84 (7)	2.13 (7)	2.961 (6)	170 (5)
N12—H12*E*⋯N9^iii^	0.97 (7)	2.56 (6)	3.125 (5)	117 (4)
N12—H12*D*⋯N9^iv^	0.91 (6)	2.24 (6)	3.125 (6)	166 (5)
N6—H6*NB*⋯N3^v^	0.89 (7)	2.59 (6)	3.251 (6)	131 (5)
N6—H6*NA*⋯N3^vi^	0.86 (7)	2.30 (7)	3.149 (6)	168 (7)

Symmetry codes: (i) 
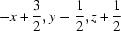
; (ii) 
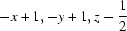
; (iii) 
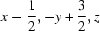
; (iv) 
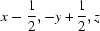
; (v) 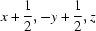; (vi) 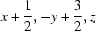.

**Fig. 1.**
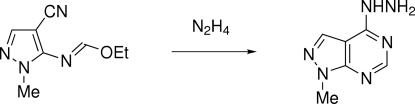
The synthesis of 4-hydrazino-1-methylpyrazolo[3,4-d]pyrimidine

**Fig. 2.**
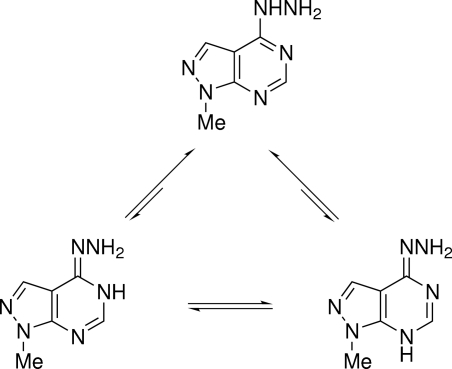
The hydrazino-hydrazono tautomerism in the title compound

**Fig. 3.**
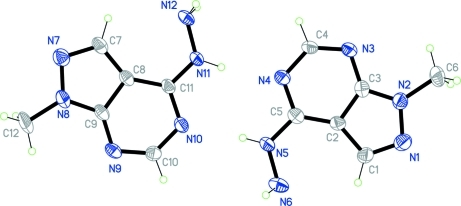
The molecular structure of 4-hydrazino-1-methylpyrazolo[3,4-d]pyrimidine with the atomic numbering scheme. Displacement ellipsoids are drawn at the 50% probability level.

**Fig. 4.**
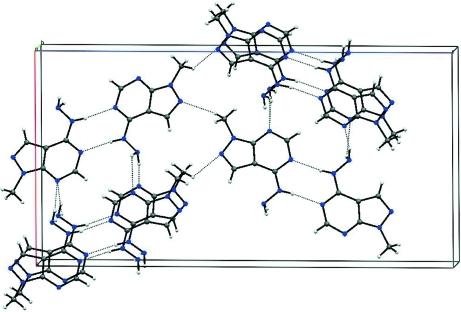
Molecular parking in the crystal, viewed along the b axis.

## supplementary crystallographic information

### Comment

The pyrazolo[3,4-*d*]pyrimidine heterocyclic system is known to be a bioisoster of purine. Therefore, chemistry of pyrazolo[3,4-*d*]pyrimidines has received significant attention, particularly for the development of new biologically active substances (Caravatti *et al.*, 2001; Dang, 2002; Schenone *et al.*, 2007; Schenone *et al.*, 2008).

Herein, we report the crystal structure of 4-hydrazino-1-methylpyrazolo[3,4-*d*]pyrimidine, which was prepared *via* a reaction of 4-cyano-5-[(ethoxymethylene)amino]-1-methylpyrazole with hydrazine according to Taylor and Loeffler (1960) (Fig. 1). Theoretically, the compound might be involved in tautomerism with three tautomeric forms (Fig. 2). However, only one tautomeric form, similarly to previously reported 1-methyl-4-(2-methylhydrazino)pyrazolo[3,4-*d*]pyrimidine (Hosmane *et al.*, 1988), was found in the crystal.

The title compound crystallizes in asymmetric unit as a dimer of two almost identical molecules (Fig. 3). Both molecules are essentially planar except for the hydrazino groups, which are turned towards pyrazole ring making the torsion angles C2—C5—N5—N6 and C8—C11—N11—N12 equal to 4.1 (7)° and 4.0 (7)°, respectively. The geometry of the molecule as well as 1.348 (6) Å and 1.332 (5) Å distances of C5—N5 and C11—N11 bonds indicate delocalization of the electron pairs of N5 and N11 with the pyrazolo[3,4-*d*]pyrimidine aromatic system.

In the crystal, the dimer molecules are linked in a *R*^2^_2_(8) graph-set motif (Bernstein *et al.*, 1995) by the N—H···N hydrogen bonds (Fig. 4, Table 1). The dimers are arranged into chains *via* intermolecular N—H···N hydrogen bonds between the hydrazino groups and the nitrogen atoms of the pyrimidine rings of both type of the molecules linking them with symmetry-related molecules in a *C*(7) graph-set motif along the [100] direction. The interactions between pyrimidine rings and hydrazino groups make a *R*^4^_4_(14) hydrogen bond motif of the fourth order. The methyl groups and the nitrogen atoms of the pyrazole rings form weak C—H···N hydrogen bonds connecting chains of the dimers in a *C*(4) motif parallel to the [100]) direction.

### Experimental

4-Hydrazino-1-methylpyrazolo[3,4-*d*]pyrimidine was synthesized by cyclocondensation of 4-cyano-5-[(ethoxymethylene)amino]-1-methylpyrazole with hydrazine according to the procedure reported by Taylor and Loeffler (1960). Single crystals suitable for crystallographic analysis were grown by recrystallization from ethanol.

### Refinement

All the H atoms attached to the carbon atoms were constrained in a riding motion approximation [0.94 Å for C_aryl_—H and 0.97 Å for methyl groups; *U*_iso_(H) =1.2*U*_eq_(C_aryl_) and *U*_iso_(H) =1.5*U*_eq_(C_methyl_)] while the N-bound H atoms were located in a difference map and refined freely. Friedel pairs were merged.

### Crystal data

**Table:**

C_6_H_8_N_6_	*D*_x_ = 1.465 Mg m^−^^3^
*M**_r_* = 164.18	Melting point: 514 K
Orthorhombic, *P**n**a*2_1_	Mo *K*α radiation, λ = 0.71073 Å
Hall symbol: P 2c -2n	Cell parameters from 3657 reflections
*a* = 14.086 (4) Å	θ = 2.9–27.2°
*b* = 3.8756 (12) Å	µ = 0.10 mm^−^^1^
*c* = 27.271 (8) Å	*T* = 223 K
*V* = 1488.8 (8) Å^3^	Block, colourless
*Z* = 8	0.58 × 0.26 × 0.10 mm
*F*(000) = 688	

### Data collection

**Table:**

Bruker SMART APEX CCD diffractometer	1715 independent reflections
Radiation source: fine-focus sealed tube	1652 reflections with *I* > 2σ(*I*)
graphite	*R*_int_ = 0.051
φ and ω scans	θ_max_ = 27.5°, θ_min_ = 1.5°
Absorption correction: multi-scan (*SADABS*; Sheldrick, 2001)	*h* = −18→18
*T*_min_ = 0.943, *T*_max_ = 0.990	*k* = −5→4
8939 measured reflections	*l* = −24→35

### Refinement

**Table:**

Refinement on *F*^2^	Primary atom site location: structure-invariant direct methods
Least-squares matrix: full	Secondary atom site location: difference Fourier map
*R*[*F*^2^ > 2σ(*F*^2^)] = 0.065	Hydrogen site location: inferred from neighbouring sites
*wR*(*F*^2^) = 0.159	H atoms treated by a mixture of independent and constrained refinement
*S* = 1.21	*w* = 1/[σ^2^(*F*_o_^2^) + (0.0699*P*)^2^ + 1.3392*P*] where *P* = (*F*_o_^2^ + 2*F*_c_^2^)/3
1715 reflections	(Δ/σ)_max_ < 0.001
243 parameters	Δρ_max_ = 0.29 e Å^−^^3^
1 restraint	Δρ_min_ = −0.28 e Å^−^^3^

### Special details

**Table:**

Geometry. All e.s.d.'s (except the e.s.d. in the dihedral angle between two l.s. planes) are estimated using the full covariance matrix. The cell e.s.d.'s are taken into account individually in the estimation of e.s.d.'s in distances, angles and torsion angles; correlations between e.s.d.'s in cell parameters are only used when they are defined by crystal symmetry. An approximate (isotropic) treatment of cell e.s.d.'s is used for estimating e.s.d.'s involving l.s. planes.
Refinement. Refinement of *F*^2^ against ALL reflections. The weighted *R*-factor *wR* and goodness of fit *S* are based on *F*^2^, conventional *R*-factors *R* are based on *F*, with *F* set to zero for negative *F*^2^. The threshold expression of *F*^2^ > σ(*F*^2^) is used only for calculating *R*-factors(gt) *etc*. and is not relevant to the choice of reflections for refinement. *R*-factors based on *F*^2^ are statistically about twice as large as those based on *F*, and *R*- factors based on ALL data will be even larger.

### Fractional atomic coordinates and isotropic or equivalent isotropic displacement parameters (Å^2^)

**Table:**

	*x*	*y*	*z*	*U*_iso_*/*U*_eq_	
N1	0.4891 (3)	0.4783 (12)	0.43889 (16)	0.0351 (9)	
N2	0.4203 (3)	0.6408 (11)	0.46578 (15)	0.0307 (9)	
N3	0.3922 (2)	0.8124 (10)	0.54938 (15)	0.0273 (9)	
N4	0.5189 (3)	0.6416 (10)	0.60298 (15)	0.0281 (8)	
N5	0.6553 (3)	0.3740 (12)	0.57903 (17)	0.0321 (9)	
H5N	0.668 (4)	0.343 (14)	0.609 (2)	0.030 (14)*	
N6	0.7119 (3)	0.2094 (13)	0.54313 (18)	0.0348 (10)	
H6NA	0.764 (5)	0.32 (2)	0.549 (3)	0.05 (2)*	
H6NB	0.731 (4)	0.007 (19)	0.555 (2)	0.037 (16)*	
N7	0.7580 (3)	0.6324 (11)	0.84251 (15)	0.0335 (9)	
N8	0.8271 (3)	0.4576 (10)	0.81702 (14)	0.0297 (9)	
N9	0.8504 (2)	0.2236 (10)	0.73690 (15)	0.0274 (9)	
N10	0.7183 (3)	0.3347 (11)	0.68245 (15)	0.0281 (8)	
N11	0.5809 (2)	0.5994 (12)	0.70605 (16)	0.0300 (9)	
H11N	0.566 (4)	0.587 (14)	0.676 (2)	0.025 (13)*	
N12	0.5243 (3)	0.7763 (12)	0.74061 (17)	0.0292 (9)	
H12D	0.469 (4)	0.656 (15)	0.743 (2)	0.030 (14)*	
H12E	0.518 (4)	1.019 (18)	0.732 (2)	0.037 (15)*	
C1	0.5567 (3)	0.3971 (14)	0.47002 (17)	0.0310 (10)	
H1	0.6128	0.2804	0.4616	0.037*	
C2	0.5337 (3)	0.5087 (12)	0.51798 (17)	0.0242 (9)	
C3	0.4448 (3)	0.6664 (12)	0.51286 (17)	0.0247 (9)	
C4	0.4345 (3)	0.7879 (13)	0.59211 (18)	0.0280 (10)	
H4	0.4016	0.8853	0.6187	0.034*	
C5	0.5701 (3)	0.5040 (12)	0.56624 (17)	0.0253 (9)	
C6	0.3354 (4)	0.7825 (17)	0.4417 (2)	0.0425 (13)	
H6A	0.3540	0.9659	0.4195	0.064*	
H6B	0.2925	0.8736	0.4663	0.064*	
H6C	0.3038	0.6013	0.4234	0.064*	
C9	0.7999 (3)	0.3896 (12)	0.77082 (18)	0.0242 (9)	
C7	0.6873 (3)	0.6765 (13)	0.81121 (18)	0.0309 (10)	
H7	0.6305	0.7923	0.8187	0.037*	
C8	0.7076 (3)	0.5295 (12)	0.76570 (18)	0.0239 (9)	
C10	0.8039 (3)	0.2142 (13)	0.69443 (18)	0.0282 (10)	
H10	0.8367	0.1051	0.6687	0.034*	
C11	0.6678 (3)	0.4953 (12)	0.71848 (16)	0.0242 (9)	
C12	0.9129 (4)	0.3412 (17)	0.8420 (2)	0.0430 (14)	
H12A	0.9554	0.2352	0.8184	0.065*	
H12B	0.8962	0.1739	0.8670	0.065*	
H12C	0.9442	0.5369	0.8571	0.065*	

### Atomic displacement parameters (Å^2^)

**Table:**

	*U*^11^	*U*^22^	*U*^33^	*U*^12^	*U*^13^	*U*^23^
N1	0.0288 (19)	0.046 (2)	0.031 (2)	−0.0051 (18)	0.0019 (17)	−0.0057 (19)
N2	0.0226 (18)	0.042 (2)	0.027 (2)	−0.0047 (16)	−0.0017 (15)	0.0028 (18)
N3	0.0193 (16)	0.030 (2)	0.032 (2)	0.0040 (15)	−0.0007 (16)	−0.0014 (16)
N4	0.0202 (18)	0.034 (2)	0.030 (2)	−0.0008 (15)	−0.0005 (14)	−0.0005 (17)
N5	0.0208 (17)	0.046 (3)	0.029 (2)	0.0086 (17)	−0.0004 (16)	0.0020 (19)
N6	0.0206 (19)	0.036 (3)	0.048 (3)	0.0011 (18)	0.0044 (18)	−0.001 (2)
N7	0.0301 (19)	0.039 (2)	0.031 (2)	0.0009 (18)	−0.0001 (16)	0.0056 (18)
N8	0.0227 (17)	0.035 (2)	0.031 (2)	−0.0009 (15)	−0.0055 (16)	0.0078 (17)
N9	0.0163 (16)	0.0300 (19)	0.036 (2)	−0.0027 (15)	0.0012 (15)	0.0041 (17)
N10	0.0222 (17)	0.035 (2)	0.0272 (19)	0.0039 (15)	−0.0023 (15)	0.0049 (17)
N11	0.0158 (17)	0.046 (2)	0.028 (2)	0.0042 (16)	−0.0029 (15)	−0.0013 (18)
N12	0.0126 (16)	0.036 (2)	0.039 (2)	0.0009 (16)	−0.0018 (15)	−0.0010 (19)
C1	0.023 (2)	0.043 (3)	0.027 (2)	−0.005 (2)	0.0039 (19)	0.002 (2)
C2	0.0166 (18)	0.028 (2)	0.029 (2)	−0.0044 (17)	0.0037 (16)	0.0003 (18)
C3	0.020 (2)	0.025 (2)	0.029 (2)	−0.0086 (16)	−0.0021 (17)	0.0052 (18)
C4	0.0210 (19)	0.035 (3)	0.029 (2)	0.0055 (18)	0.0048 (18)	0.0001 (18)
C5	0.0191 (17)	0.024 (2)	0.033 (2)	−0.0056 (15)	0.0011 (16)	0.0043 (17)
C6	0.032 (3)	0.058 (4)	0.037 (3)	0.001 (2)	−0.015 (2)	−0.001 (3)
C9	0.0162 (17)	0.023 (2)	0.033 (2)	−0.0034 (16)	−0.0032 (17)	0.0062 (18)
C7	0.0192 (19)	0.039 (3)	0.035 (2)	−0.0018 (18)	−0.0007 (19)	0.006 (2)
C8	0.0158 (17)	0.025 (2)	0.031 (2)	−0.0027 (15)	0.0003 (17)	0.0072 (18)
C10	0.022 (2)	0.035 (2)	0.028 (2)	−0.0029 (18)	0.0048 (18)	0.0010 (19)
C11	0.0145 (17)	0.031 (2)	0.027 (2)	−0.0012 (16)	−0.0001 (16)	0.0029 (18)
C12	0.029 (2)	0.056 (3)	0.044 (3)	0.005 (2)	−0.018 (2)	0.010 (3)

### Geometric parameters (Å, °)

**Table:**

N1—C1	1.314 (7)	N11—C11	1.332 (5)
N1—N2	1.369 (6)	N11—N12	1.412 (6)
N2—C3	1.333 (6)	N11—H11N	0.85 (6)
N2—C6	1.470 (6)	N12—H12D	0.91 (6)
N3—C4	1.312 (6)	N12—H12E	0.97 (7)
N3—C3	1.364 (6)	C1—C2	1.415 (7)
N4—C5	1.345 (6)	C1—H1	0.9400
N4—C4	1.350 (6)	C2—C3	1.401 (6)
N5—C5	1.348 (6)	C2—C5	1.413 (6)
N5—N6	1.415 (6)	C4—H4	0.9400
N5—H5N	0.84 (7)	C6—H6A	0.9700
N6—H6NA	0.86 (7)	C6—H6B	0.9700
N6—H6NB	0.89 (7)	C6—H6C	0.9700
N7—C7	1.322 (6)	C9—C8	1.415 (5)
N7—N8	1.375 (6)	C7—C8	1.395 (7)
N8—C9	1.343 (6)	C7—H7	0.9400
N8—C12	1.458 (6)	C8—C11	1.411 (6)
N9—C10	1.331 (6)	C10—H10	0.9400
N9—C9	1.332 (6)	C12—H12A	0.9700
N10—C10	1.334 (6)	C12—H12B	0.9700
N10—C11	1.364 (6)	C12—H12C	0.9700
			
C1—N1—N2	106.1 (4)	N3—C4—N4	128.7 (4)
C3—N2—N1	111.5 (4)	N3—C4—H4	115.7
C3—N2—C6	127.8 (4)	N4—C4—H4	115.7
N1—N2—C6	120.5 (4)	N4—C5—N5	115.7 (4)
C4—N3—C3	111.9 (4)	N4—C5—C2	119.6 (4)
C5—N4—C4	118.4 (4)	N5—C5—C2	124.7 (4)
C5—N5—N6	119.4 (4)	N2—C6—H6A	109.5
C5—N5—H5N	120 (4)	N2—C6—H6B	109.5
N6—N5—H5N	119 (4)	H6A—C6—H6B	109.5
N5—N6—H6NA	97 (5)	N2—C6—H6C	109.5
N5—N6—H6NB	108 (4)	H6A—C6—H6C	109.5
H6NA—N6—H6NB	97 (6)	H6B—C6—H6C	109.5
C7—N7—N8	105.7 (4)	N9—C9—N8	126.4 (4)
C9—N8—N7	111.6 (4)	N9—C9—C8	127.3 (4)
C9—N8—C12	127.8 (4)	N8—C9—C8	106.2 (4)
N7—N8—C12	120.2 (4)	N7—C7—C8	111.6 (4)
C10—N9—C9	110.8 (4)	N7—C7—H7	124.2
C10—N10—C11	117.1 (4)	C8—C7—H7	124.2
C11—N11—N12	119.7 (4)	C7—C8—C11	140.2 (4)
C11—N11—H11N	118 (3)	C7—C8—C9	104.9 (4)
N12—N11—H11N	122 (4)	C11—C8—C9	114.8 (4)
N11—N12—H12D	107 (4)	N9—C10—N10	130.4 (5)
N11—N12—H12E	111 (4)	N9—C10—H10	114.8
H12D—N12—H12E	116 (5)	N10—C10—H10	114.8
N1—C1—C2	111.0 (4)	N11—C11—N10	115.7 (4)
N1—C1—H1	124.5	N11—C11—C8	124.7 (4)
C2—C1—H1	124.5	N10—C11—C8	119.5 (4)
C3—C2—C5	115.0 (4)	N8—C12—H12A	109.5
C3—C2—C1	104.2 (4)	N8—C12—H12B	109.5
C5—C2—C1	140.7 (4)	H12A—C12—H12B	109.5
N2—C3—N3	126.4 (4)	N8—C12—H12C	109.5
N2—C3—C2	107.2 (4)	H12A—C12—H12C	109.5
N3—C3—C2	126.4 (4)	H12B—C12—H12C	109.5
			
C1—N1—N2—C3	1.0 (5)	C3—C2—C5—N5	−178.1 (4)
C1—N1—N2—C6	177.0 (4)	C1—C2—C5—N5	3.4 (9)
C7—N7—N8—C9	−0.8 (5)	C10—N9—C9—N8	−177.2 (4)
C7—N7—N8—C12	−175.0 (4)	C10—N9—C9—C8	2.7 (6)
N2—N1—C1—C2	−0.6 (6)	N7—N8—C9—N9	−179.8 (4)
N1—C1—C2—C3	0.1 (6)	C12—N8—C9—N9	−6.0 (7)
N1—C1—C2—C5	178.6 (5)	N7—N8—C9—C8	0.3 (5)
N1—N2—C3—N3	−179.7 (4)	C12—N8—C9—C8	174.0 (5)
C6—N2—C3—N3	4.7 (8)	N8—N7—C7—C8	1.0 (6)
N1—N2—C3—C2	−0.9 (5)	N7—C7—C8—C11	−177.1 (5)
C6—N2—C3—C2	−176.6 (5)	N7—C7—C8—C9	−0.8 (6)
C4—N3—C3—N2	178.0 (4)	N9—C9—C8—C7	−179.7 (4)
C4—N3—C3—C2	−0.5 (6)	N8—C9—C8—C7	0.3 (5)
C5—C2—C3—N2	−178.5 (4)	N9—C9—C8—C11	−2.3 (7)
C1—C2—C3—N2	0.5 (5)	N8—C9—C8—C11	177.7 (4)
C5—C2—C3—N3	0.3 (6)	C9—N9—C10—N10	−1.5 (7)
C1—C2—C3—N3	179.3 (4)	C11—N10—C10—N9	0.0 (7)
C3—N3—C4—N4	−0.3 (7)	N12—N11—C11—N10	−178.3 (4)
C5—N4—C4—N3	1.3 (8)	N12—N11—C11—C8	4.0 (7)
C4—N4—C5—N5	177.5 (4)	C10—N10—C11—N11	−177.1 (4)
C4—N4—C5—C2	−1.5 (6)	C10—N10—C11—C8	0.6 (6)
N6—N5—C5—N4	176.9 (4)	C7—C8—C11—N11	−6.0 (9)
N6—N5—C5—C2	−4.1 (7)	C9—C8—C11—N11	177.9 (4)
C3—C2—C5—N4	0.8 (6)	C7—C8—C11—N10	176.4 (5)
C1—C2—C5—N4	−177.7 (6)	C9—C8—C11—N10	0.4 (6)

### Hydrogen-bond geometry (Å, °)

**Table:**

*D*—H···*A*	*D*—H	H···*A*	*D*···*A*	*D*—H···*A*
C12—H12B···N1^i^	0.97	2.65	3.297 (7)	124
C6—H6C···N7^ii^	0.97	2.54	3.410 (7)	150
N11—H11N···N4	0.85 (6)	2.11 (6)	2.948 (6)	170 (5)
N5—H5N···N10	0.84 (7)	2.13 (7)	2.961 (6)	170 (5)
N12—H12E···N9^iii^	0.97 (7)	2.56 (6)	3.125 (5)	117 (4)
N12—H12D···N9^iv^	0.91 (6)	2.24 (6)	3.125 (6)	166 (5)
N6—H6NB···N3^v^	0.89 (7)	2.59 (6)	3.251 (6)	131 (5)
N6—H6NA···N3^vi^	0.86 (7)	2.30 (7)	3.149 (6)	168 (7)

Symmetry codes: (i) −*x*+3/2, *y*−1/2, *z*+1/2; (ii) −*x*+1, −*y*+1, *z*−1/2; (iii) *x*−1/2, −*y*+3/2, *z*; (iv) *x*−1/2, −*y*+1/2, *z*; (v) *x*+1/2, −*y*+1/2, *z*; (vi) *x*+1/2, −*y*+3/2, *z*.
